# Hypertriglyceridemia as a possible cause of coma: a case report

**DOI:** 10.1186/1752-1947-6-412

**Published:** 2012-11-30

**Authors:** Ryota Inokuchi, Akinori Matsumoto, Ryuta Azihara, Hajime Sato, Yoshibumi Kumada, Hideyuki Yokoyama, Megumi Okada, Tokiya Ishida, Kensuke Nakamura, Susumu Nakajima, Naoki Yahagi, Kazuaki Shinohara

**Affiliations:** 1 Department of Emergency and Critical Care Medicine, Ohta Nishinouchi Hospital, 2-5-20 Nishinouchi, Koriyama, Fukushima, 963-8558, Japan; 2 Department of Emergency and Critical Care Medicine, The University of Tokyo Hospital, 7-3-1 Hongo, Bunkyo-ku, Tokyo, 113-8655, Japan; 3 Department of Health Policy and Technology Assessment, National Institute of Public Health, 2-3-6 Minami, Wako, Saitama, 351-0197, Japan

**Keywords:** Hypertriglyceridemia, Plasma viscosity, Alcohol, Diabetes mellitus, Transient ischemic attack

## Abstract

**Introduction:**

Many studies suggest that elevated triglyceride levels are associated with increased long-term risk of stroke, including transient ischemic attacks. In addition, elevated triglyceride levels independently contribute to plasma viscosity and decreased blood flow. However, no consensus has been reached regarding the significance of hypertriglyceridemia as an independent risk factor for ischemic stroke.

**Case presentation:**

We report the case of a patient admitted to our hospital for sudden onset of coma. Laboratory test results revealed he had high blood glucose (28.2mmol/L), high glycated hemoglobin (11.4 percent), considerably high serum triglyceride levels (171.5mmol/L; type V hyperlipoproteinemia), and high plasma viscosity (1.90mPa/s) with normal β-hydroxybutyric acid levels. His triglyceride levels decreased after administering intravenous fluids. Our patient’s consciousness level improved gradually over three days. All serum lipid levels decreased seven days after admission.

**Conclusions:**

The findings in our patient’s case are likely explained by triglyceride-mediated hyperviscosity causing a transient ischemic attack. In the present report we suggest that when several tests do not reveal the cause of stroke-like symptoms, measurement of plasma viscosity may be informative.

## Introduction

Elevated triglyceride levels are associated with increased long-term risk of ischemic stroke [1]. Short-term complications include pancreatitis, which is an emergent problem, and other complications that have been reported in familial hypertriglyceridemia, including recent memory loss, abdominal pain, dyspnea, eruptive xanthoma, flushing after alcohol consumption, and lipemia retinalis [2]. However, no consensus has been reached regarding the significance of hypertriglyceridemia as an independent risk factor for ischemic stroke [3]. Here, we report the case of a patient with sudden coma likely caused by triglyceride-mediated hyperviscosity.

## Case presentation

A comatose 56-year-old Japanese man with no significant familial medical history was admitted to the emergency room. He delivered newspapers and usually consumed alcohol two hours before delivery. According to colleagues, during his usual morning routine, he suddenly fell on his back. On admission, his temperature was 36.0°C, pulse rate 77 beats/min, blood pressure 153/94mmHg and Glasgow Coma Scale score was E2V2M1. On examination, splenohepatomegaly was detected, but heart murmur, chest rales, tongue biting, incontinence, diaphoresis, seizure, and xanthoma were not evident. He maintained a balanced diet and was not obese (height 164cm, weight 58.8kg and body mass index 21.9kg/m^2^).

**Figure 1:**
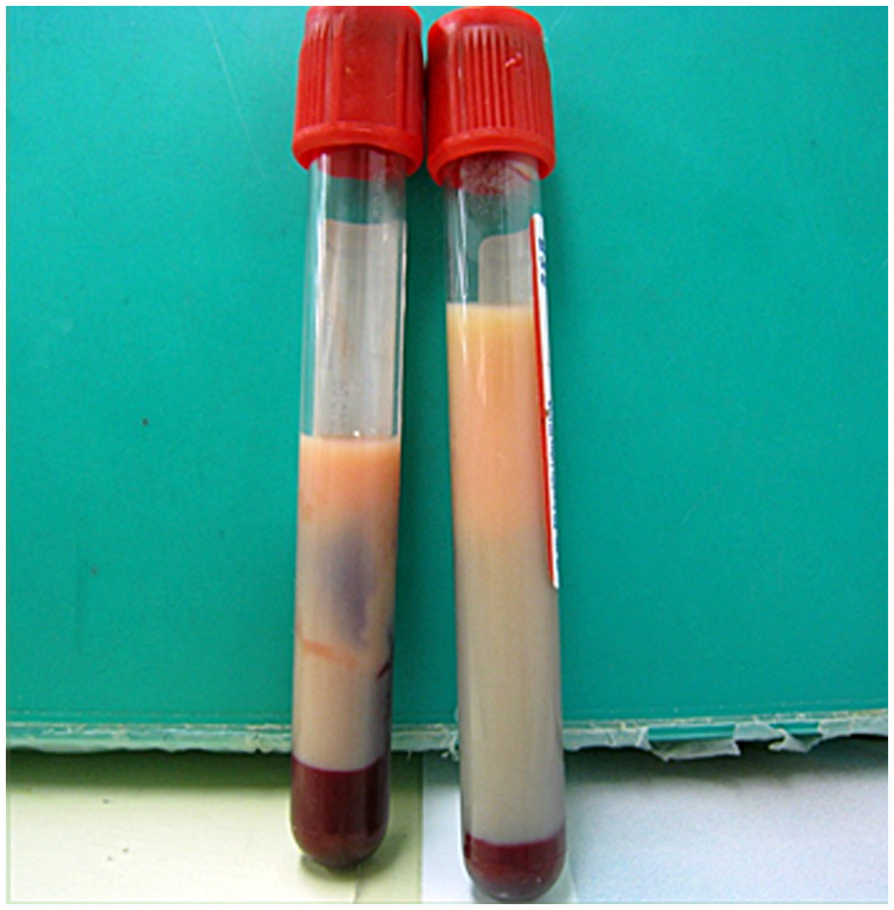
**Milky plasma.** Our patient’s plasma had a milky appearance.

**Figure 2:**
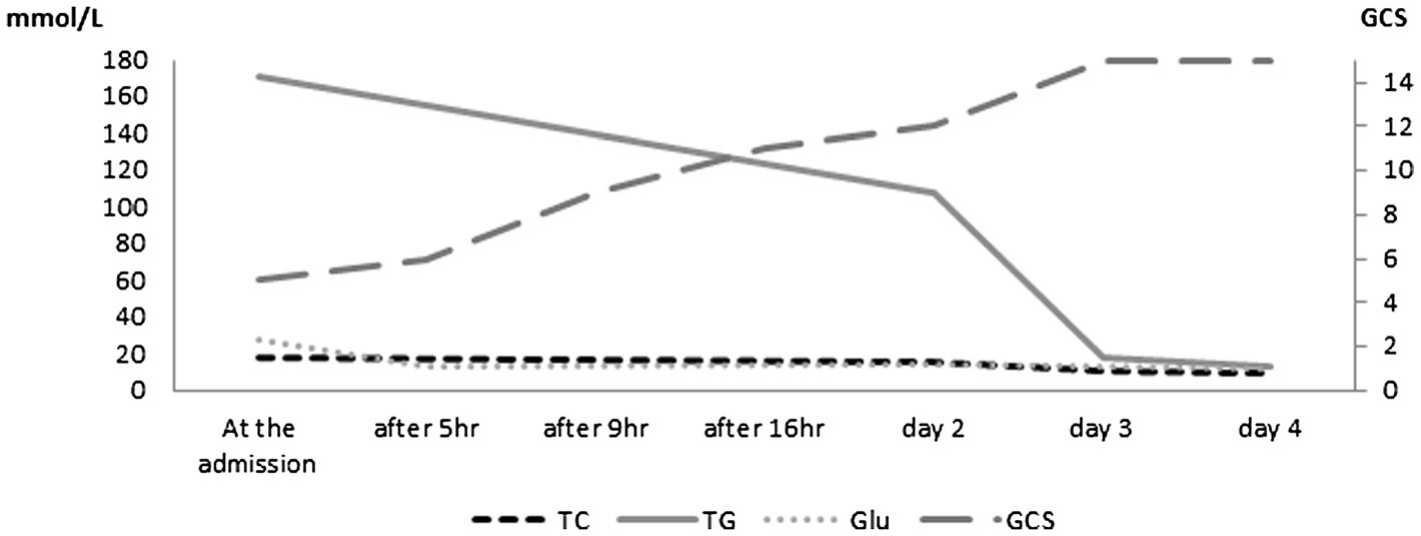
**Treatment course.** After administration of intravenous fluids, as our patient’s serum triglyceride level decreased, his consciousness level gradually improved.

Arrhythmia was not detected on admission. Laboratory test results revealed high blood glucose (28.2mmol/L), high glycated hemoglobin (11.4 percent), considerably high serum lipid levels (triglycerides 171.5mmol/L, total cholesterol (T-Chol) 17.7mmol/L, high-density lipoprotein (HDL) 0.5mmol/L, low-density lipoprotein (LDL) 1.9mmol/L and type V hyperlipoproteinemia (Figure 1), and slightly elevated liver enzyme levels. Serum osmotic pressure was 356mOsm/L, and alcohol concentration was 15.1mmol/L. His white blood cell count, electrolyte levels (Mg, inorganic phosphorous, and Ca), C-reactive protein level, renal function, NH_3_ level, coagulation (prothrombin time/international normalized ratio, activated partial thromboplastin time, fibrinogen, fibrin/fibrinogen degradation products), thyroid function, urine drug levels, vitamin levels, pyruvic acid level, and β-hydroxybutyric acid level were normal. Venous blood gas was almost normal (pH 7.389, pCO_2_ 38.7mmHg, pO_2_ 102.6mmHg, HCO_3_ 22.8mmol/L, anion gap 14.3 and base excess −1.8mmol/L). Plasma viscosity was 1.90mPa/s (normal range 1.10 to 1.30mPa/s at 37°C; Lovis 2000 M/ME, Anton Paar, Graz, Austria), as observed later. Results of electrocardiography, chest radiography, brain computed tomography (CT), chest and abdominal contrast-enhanced CT, diffusion-weighted magnetic resonance imaging, magnetic resonance angiography including examination of the cervical internal carotid arteries, electroencephalography, and auditory brainstem response testing were normal. Cultures of blood, urine, and cerebrospinal fluid were negative. Fundus examination was not performed. Hyperglycemia immediately decreased following isotonic saline infusion with no insulin therapy. By contrast, triglyceride levels gradually decreased after administration of intravenous fluids. Our patient’s consciousness level improved gradually over three days (Figure 2). All serum lipid levels decreased seven days after admission (triglycerides 12.8mmol/L, T-Chol 9.5mmol/L, HDL 0.7mmol/L, and LDL 6.3mmol/L).

## Discussion

We report the case of a patient with sudden coma likely caused by triglyceride-mediated hyperviscosity. Hypertriglyceridemia may lead to ischemic stroke through its contribution to atherosclerosis and/or thrombogenicity. Studies suggest that hypertriglyceridemia fosters the development of atherosclerosis via several mechanisms, namely promotion of atherosclerosis, endothelial dysfunction, oxidative stress due to lipid-derived free radicals, impairment of endothelial-dependent vasodilatation, association with elevated atherosclerosis markers (C-reactive protein, fibrinogen levels and circulating adhesion molecules), promotion of thrombogenicity, elevated plasma viscosity, elevated plasma fibrinogen levels, lowered fibrinolytic activity, elevated levels of clotting factor Xc compared to normolipidemic controls, and elevated fibrinogen levels [3]. Among them, Rosenson *et al*. reported that elevated triglyceride levels independently contribute to plasma viscosity and decrease blood flow [4], and Tomiyama *et al*. reported that plasma viscosity is associated with cerebral blood flow [5]. Following high-dose intravenous immunoglobulin therapy, blood and plasma viscosity can increase [6], and the risk of thromboembolism (including stroke) increases when serum viscosity exceeds a threshold after high-dose intravenous immunoglobulin therapy [7]. Therefore, hypertriglyceridemia may increase the risk of stroke by inducing a prothrombotic state through its effects on plasma viscosity.

Hypertriglyceridemia can occur because of obesity, poorly controlled diabetes mellitus, alcohol misuse, and familial disease. In our patient’s case, he had no family history, and therefore, the causes of hypertriglyceridemia were likely to be poor control of diabetes mellitus and alcohol misuse. Many case reports and series have described apheresis for hypertriglyceridemia. However, we did not pursue this treatment because our patient responded to fluid therapy and did not exhibit pancreatitis.

The effects of alcohol and alcohol withdrawal cannot be ruled out in our patient. However, people with chronic alcoholism may demonstrate little clinical evidence of intoxication even with blood alcohol levels >22.2mmol/L [8]. In addition, alcohol withdrawal cannot explain the symptoms, because withdrawal symptoms typically begin four to 12 hours after alcohol cessation. Our patient’s symptoms appeared two hours after consumption, and his vital signs and physical characteristics did not indicate alcohol withdrawal. With regard to hyperglycemia, symptoms of hyperosmolar hyperglycemic state develop insidiously with polyuria, polydipsia, and weight loss, often persisting for several days before hospital admission, and diabetic ketoacidosis usually evolves more rapidly, over a 24-hour period. In our patient’s case, his symptoms developed too quickly and his β-hydroxybutyric acid level was normal. Therefore, the probability of hyperglycemia being the direct cause of his coma is low.

## Conclusions

We report the case of a patient who entered coma, likely caused by triglyceride-mediated hyperviscosity causing a transient ischemic attack. We suggest that when several tests do not reveal the cause of stroke-like symptoms, measurement of plasma viscosity may be informative.

## Consent

Written informed consent was obtained from the patient for publication of this case report and any accompanying images. A copy of the written consent is available for review by the Editor-in-Chief of this journal.

## Competing interests

The authors declare that they have no competing interests.

## Authors’ contributions

RI, AM, RA, KS, HY, MO, and TI contributed to patient management. RI, KS, NK, SN, and NY contributed to writing and reviewing the report. RI and NK measured plasma viscosity. All authors read and approved the final manuscript.
